# Oncogene APOL1 promotes proliferation and inhibits apoptosis via activating NOTCH1 signaling pathway in pancreatic cancer

**DOI:** 10.1038/s41419-021-03985-1

**Published:** 2021-08-02

**Authors:** Jiewei Lin, Zhiwei Xu, Junjie Xie, Xiaxing Deng, Lingxi Jiang, Hao Chen, Chenghong Peng, Hongwei Li, Jiaqiang Zhang, Baiyong Shen

**Affiliations:** 1 Pancreatic Disease Center, Department of General Surgery, Ruijin Hospital, Shanghai Jiao Tong University School of Medicine, Shanghai, China; 2 Research Institute of Pancreatic Diseases, Shanghai Jiao Tong University School of Medicine, Shanghai, China; 3 State Key Laboratory of Oncogenes and Related Genes, Shanghai, China; 4 Institute of Translational Medicine, Shanghai Jiao Tong University, Shanghai, China

**Keywords:** Oncogenes, Protein-protein interaction networks

## Abstract

APOL1 encodes a secreted high-density lipoprotein, which has been considered as an aberrantly expressed gene in multiple cancers. Nevertheless, the role of APOL1 in the regulatory mechanisms of pancreatic cancer remains unknown and should be explored. We identified APOL1 was abnormally elevated in human pancreatic cancer tissues compared with that in adjacent tissues and was associated with poor prognosis. The effects of APOL1 in PC cell proliferation, cell cycle, and apoptosis was verified via functional in vitro and in vivo experiments. The results showed that knockdown of APOL1 significantly inhibited the proliferation and promoted apoptosis of pancreatic cancer. In addition, we identified APOL1 could be a regulator of NOTCH1 signaling pathway using bioinformatics tools, qRT-PCR, dual-luciferase reporter assay, and western blotting. In summary, APOL1 could function as an oncogene to promote proliferation and inhibit apoptosis through activating NOTCH1 signaling pathway expression in pancreatic cancer; therefore, it may act as a novel therapeutic target for pancreatic cancer.

## Introduction

Pancreatic cancer (PC) is the most aggressive fatal cancer of the alimentary tract and it remains difficult to diagnose with a grave prognosis, high mortality, and a 5-year survival rate of <5% [1]. The treatment choices for PC are limited; for some, the only potential cure is radical surgery [2]. Chemotherapy is the only option for advanced patients despite wide resistance [3]. Few functional targets of PC and their underlying mechanisms have been established. Thus, it is imperative to identify the underlying molecular mechanisms of PC occurrence and development, and to develop novel therapeutic targets.

Apolipoproteins can combine with lipids to constitute lipoproteins, which shuttle lipids through systemic circulation [4]. Numerous studies have reported that apolipoproteins are related to various biotic processes, including inflammatory response, immunoreactions, and tumor progression [5–7]. Recent studies have shown that apolipoproteins can act as biomarkers for early diagnosis of several tumors and as prognostic biomarkers, and are directly involved in cell growth, apoptosis, cell cycle, migration, and invasion in multiple tumors [8, 9].

Apolipoprotein L1 (APOL1) is a secreted high-density lipoprotein (HDL) that can bind to apolipoprotein A1 (APOA1), which participates in lipid transport and metabolism [10]. Elevated APOA1 was detected in multiple tumors, such as hepatocellular carcinoma, small-cell lung carcinoma, and bladder cancer [11–13]. APOL1 is overexpressed in papillary thyroid carcinoma, PC, and head-and-neck squamous cell carcinoma [14–17]. Inversely, APOL1 is significantly downregulated in renal cancer tissues and cells [18]. However, the underlying mechanism of its functions in tumorigenesis and development of PC remain poorly understood.

NOTCH1, of the NOTCH receptor family (NOTCH1–4), performs vital cell functions, such as proliferation, differentiation, apoptosis, and stem cell maintenance [19]. NOTCH receptors have been verified as oncogenes in several cancer types, including colorectal cancer, cervical cancer, leukemia, lung cancer, breast cancer, and oral squamous cell carcinoma [20]. However, NOTCH1 can act as either an oncogene or anti-oncogene largely depending on PC progression [21].

Recently, bioinformatics analysis and gene expression profiling are considered as functional tools for exploring the mechanisms of tumorigenesis and development [22]. The identified key genes contribute to determining prognosis and provide a potential mechanism for cancer progression via re-analyzing and integrating public databases [23]. For instance, several significant differentially expressed genes (DEGs) involved in colorectal cancer were identified, which may act as new biomarkers or potential targets for colorectal cancer [24].

Using bioinformatics-based analysis followed by experimental validation, we found that APOL1 was significantly increased in PC tissues and was positively correlated with advanced pathological stage and poor prognosis of PC patients. Moreover, APOL1 silencing inhibited PC cell growth and induced cell cycle arrest and apoptosis in vitro, and suppressed tumor growth in vivo. Bioinformatics analysis showed that high APOL1 expression was positively correlated with NOTCH1 signaling pathway and APOL1 exerts an oncogenic role in PC via activating the NOTCH1 signaling pathway. In conclusion, our results indicated that APOL1 might be a new therapeutic target for PC.

## Methods

### Microarray data

We selected datasets GSE41368, GSE28735, and GSE62452 from the Gene Expression Omnibus (GEO) database using the GPL6244 platform (Affymetrix Human Gene 1.0 ST Array). GSE41368 included 6 pancreatic tissues and 6 non-tumor samples, GSE28735 included 45 matching pairs of pancreatic tumor tissues and non-cancerous tissues, and GSE62452 included 69 pancreatic tumor tissues and 61 adjacent non-cancerous tissues. We used GEO2R (https://www.ncbi.nlm.nih.gov/geo/geo2r/) to identify DEGs between PC tissues and non-cancerous tissues. GEO2R is an interactive online tool that allows users to potentially detect DEGs in a GEO series [25]. Log|FC| (fold change) > 1 and adjusted *p*-value < 0.01 were considered statistically significant. Venn software online (http://bioinformatics.psb.ugent.be/webtools/Venn/) was used to discover common DEGs in these three databases.

### PPI network, GO, and KEGG pathway enrichment analysis

The protein–protein interaction (PPI) network was evaluated using Search Tool for the Retrieval of Interacting Genes (STRING; http://string-db.org) [26]. The PPI network information in STRING was re-analyzed using Cytoscape software version 3.7.2 (combined score > 0.4) [27]. The MCODE application in Cytoscape was used to identify modules of the PPI network (degree cutoff = 2, max. *k*-core = 2, Depth = 100, and node score cutoff = 0.2).The Database for Annotation, Visualization, and Integrated Discovery (DAVID, https://david.ncifcrf.gov) provided tools for Gene Ontology (GO) and Kyoto Encyclopedia of Genes and Genomes (KEGG) pathway analyses.

### DEG expression and survival analysis

Gene Expression Profiling Interactive Analysis (GEPIA; http://gepia.cancer-pku.cn/index.html) was used to analyze expression levels of the hub genes and stage plot. Kaplan–Meier analysis was used to analyze overall survival (OS) and disease-free survival (DFS) of the hub genes in PC.

### Patient samples

Next, 77 pairs of PC tissues and paired normal pancreas tissues were obtained from patients at Ruijin Hospital Affiliated with Shanghai Jiao Tong University School of Medicine (Shanghai, China). All samples were snap-frozen in liquid nitrogen and stored at −80 °C until use. These patients had not received any preoperative chemotherapy treatment before surgery. Our study protocols were approved by Institutional Ethics Committees of Ruijin Hospital.

### Cell culture and transfection

Human PC cell lines (Aspc-1, Bxpc-3, CFPAC-1, MIA PaCa-2, PANC-1, and PATU-8988) and human normal pancreatic ductal epithelial (HPNE) cells were obtained from the Cell Bank of the Chinese Academy of Sciences. Cells were cultured in Dulbecco’s modified Eagle’s medium, RPMI-1640, and Iscove’s modified Dulbecco’s medium, all with 10% fetal bovine serum. Both microscopic observation and cultivation revealed that cells were free from contaminating microorganisms, including mycoplasma.

For in vitro experiments, APOL1 small interfering RNA (siRNA) (si-APOL1), NOTCH1 siRNA (si-NOTCH1), and NC siRNA (siRNA-NC) were synthesized by Biogene (Shanghai, China). Full-length APOL1 cDNA were synthesized and inserted into plasmids (Bioegene, Shanghai, China). The small hairpin RNA (shRNA) of APOL1 was synthesized and cloned into the piLenti-shRNA-GFP-Puro vector by Bioegene (Shanghai, China). PANC-1 and MIA PaCa-2 cells transduced with lentivirus were treated with 2 µg/mL puromycin for 48 h to establish stable cell lines. All transfections were performed using Lipofectamine 3000 (Invitrogen). Cells were collected at 48 h post transfection. The siRNA sequences are listed in Supplementary Table S1.

### Quantitative reverse-transcriptase PCR

Total RNA was extracted from pancreatic frozen tissues and cell lines using TRIzol Reagent (Invitrogen, Carlsbad, CA, USA). mRNA was detected using AceQ Universal SYBR qPCR Master Mix (Vazyme, Nanjing, China). ACTB expression was employed as an internal mRNA control. The primer sequences are listed in Supplementary Table S2.

### Western blotting

Proteins were extracted from PANC-1 and MIA PaCa-2 cells using RIPA buffer, loaded and run on a 10% SDS-polyacrylamide gel electrophoresis separation gel, and transferred onto a polyvinylidene difluoride membrane. The following primary antibodies were used: anti-APOL1, anti-CCND1, anti-CDK4, anti-CDK6, anti-PARP, anti-Bax, anti-NOTCH1-IC, anti-HES1, anti-HES5, anti-c-Myc, and anti-glyceraldehyde 3-phosphate dehydrogenase (GAPDH). Protein expression was detected by ECL reagents. GAPDH was used as a control. The antibodies used are listed in Supplementary Table S3.

### Immunohistochemistry

Immunohistochemistry (IHC) was performed as previously described, with a brief modification [28]. The tumor tissues excised from nude mice and patient specimens were fixed, dehydrated, and embedded in paraffin. They were then sliced into 4 μm sections and used for IHC (primary antibodies against APOL1, ki-67, terminal deoxynucleotidyl transferase biotin-dUTP nick end labeling (TUNEL), and NOTCH1-IC), followed by 30 min incubation with a biotin-labeled secondary antibody.

### Gene set enrichment analysis

Gene set enrichment analysis (GSEA) was used to elucidate APOL1-related pathways and gene sets in PC. Gene expression profiles of 179 PC samples were downloaded from The Cancer Genome Atlas (TCGA) datasets. According to APOL1 expression, the top 50% and bottom 50% of samples were grouped as high- and low-APOL1 groups, respectively. The number of permutations was 1000 and the threshold for the adjusted *p*-value was set to 0.05. If most members of a gene set were positively or negatively related to APOL1, the set was considered to be related to APOL1.

### Flow cytometric analysis

To analyze cell cycle arrest, cells were collected, fixed with 70% ethanol, and stored at 4 °C overnight. After washing with phosphate-buffered saline (PBS) twice, cells were resuspended in 400 μL of PI/RNase Staining Buffer (BD Biosciences, San Jose, CA, USA), stained for 15 min in a dark room at room temperature, and analyzed by flow cytometry. For the apoptosis assay, the PC cells were resuspended, incubated with fluorescein isothiocyanate-conjugated annexin V and propidium iodide for 30 min in the dark at room temperature, and were analyzed by flow cytometry.

### EdU, CCK-8, and colony formation assays

Cell proliferation was detected with an EdU Labeling Kit (Beyotime Biotechnology, Haimen, China). Transfected cells (2 × 10^4^ cells/well) were seeded in 24-well plates and cultured for 72 h. Subsequently, cells were incubated with EdU for 2 h, stained by 4% paraformaldehyde for 15 min, and subjected to nuclear staining using Hoechst. Transfected PC cells were plated in 96-well plates at 3 × 10^3^ cells/well using Cell Counting Kit-8 (CCK-8, Dojindo, Shanghai, China) to evaluate cell viability. Absorbance (optical density) was measured at 450 nm. For the colony formation assay, transfected PC cells were seeded in six-well plates at 1 × 10^3^ cells/plate and cultured for 14 days.

### In vivo xenograft model

Male 3- to 4-week-old BALB/c-nu nude mice (16–18 g) were purchased from Chinese Academy of Sciences (Shanghai, China). Stably transfected PANC-1 and MIA PaCa-2 cells were washed by PBS twice and injected subcutaneously into mice (5 × 10^6^ cells/site). The longest longitudinal diameter (*a*) and longest transverse diameter (*b*) were measured every 4 days from the first injection, and tumor volumes were calculated as follows: volume = 1/2 (*a* × *b*^2^). Mice were killed after 32 days and tumors were weighed.

### Dual-luciferase reporter assay

A pGL3-based construct containing HES1, HES5, or c-Myc promoter sequences was synthesized (Promega, Madison, WI, USA) and cloned into reporter plasmids. Luciferase activity was measured using the dual-luciferase reporter assay system (Promega, USA) and normalized to *Renilla* luciferase activity.

### Statistical analysis

SPSS 20.0 and GraphPad Prism 7.0 were used to perform statistical analysis. Experiments were repeated independently at least three times and presented as the mean ± SD. *P* < 0.05 was considered statistically significant. One-way analysis of variance, Student’s *t*-test, and *χ*^2^-test were used to analyze the means between groups. Survival curves were analyzed using the Kaplan–Meier method and log-rank tests were applied to evaluate differences between groups.

## Results

### Identification of significant DEGs in PC

**Fig. 1:**
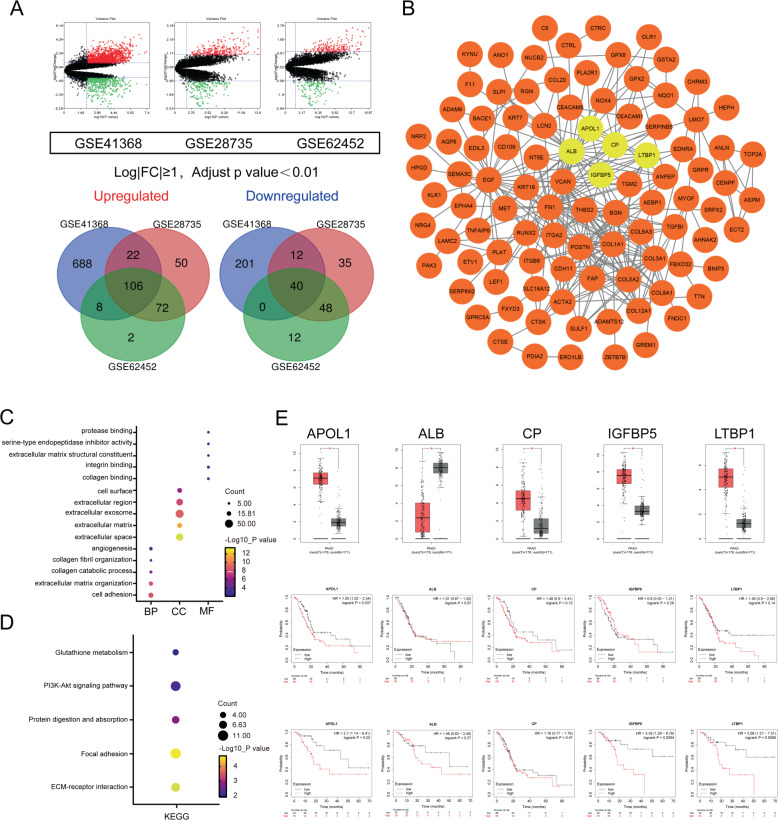
Identification of the significant DEGs in pancreatic cancer. **A** Volcano plot and intersection of the upregulated and downregulated DEGs in GSE41368, GSE28735, and GSE62452. **B** PPI network of the overlapping DEGs. Yellow circles indicate the most significant module selected from MCODE in Cytoscape. **C** GO analysis of the significant DEGs in PC. **D** KEGG pathway analysis of DEGs in PC. **E** Expression levels between tumor tissues and normal tissues of the five hub genes in GEPIA. Red indicates tumor tissues; gray represents normal tissues. OS and DFS of five hub genes were determined using Kaplan–Meier plotter databases.

To identify common DEGs in PC tissues, GSE41368, GSE28735, and GSE62452 datasets were obtained from the GEO database and comprehensively analyzed via GEO2R online tools, resulting in 1077, 385, and 288 DEGs, respectively. The overlapping DEGs among those datasets were substantiated, resulting in 146 common DEGs, including 106 upregulated and 40 downregulated genes (Fig. 1A).

Next, the DEG PPI network was constructed via the STRING network database (Supplementary Fig. S1A). The five most vital genes were detected by Cytoscape MCODE, including *APOL1*, *CP*, *LTBP1*, *ALB*, and *IGFBP5* (Fig. 1B). The 146 DEGs were analyzed by DAVID software to thoroughly examine their underlying functions. GO analysis revealed that for biological processes, the DEGs were largely enriched in the following functions: extracellular matrix organization, collagen catabolic process, collagen fibril organization, cell adhesion, and angiogenesis. For cell components, they were enriched in cell surface, extracellular space, extracellular matrix, extracellular exosome, and extracellular region. For molecular functions, they were enriched in extracellular matrix structural constituent, collagen binding, protease binding, integrin binding, and serine-type endopeptidase inhibitor activity (Fig. 1C). KEGG analysis demonstrated that DEGs were significantly enriched in glutathione metabolism, ECM–receptor interaction, focal adhesion, protein digestion and absorption, and the PI3K-Akt signaling pathway (Fig. 1D). We input five hub genes in GEPIA to analyze expression between cancerous and paired non-tumor tissues. Expression of APOL1, CP, IGFBP5, and LTBP1 was upregulated and ALB was downregulated in cancerous tissues compared to those in paired normal tissues. Kaplan–Meier plotter (http://kmplot.com/analysis) was used to examine associations between the five hub genes and survival. We found that only upregulated APOL1 was related to worse OS and DFS, which may play an imperative role in PC (Fig. 1E). Therefore, we examined the role of APOL1 in PC in subsequent experiments.

### APOL1 is significantly upregulated in PC and is related to later pathological stage and poor prognosis

**Fig. 2:**
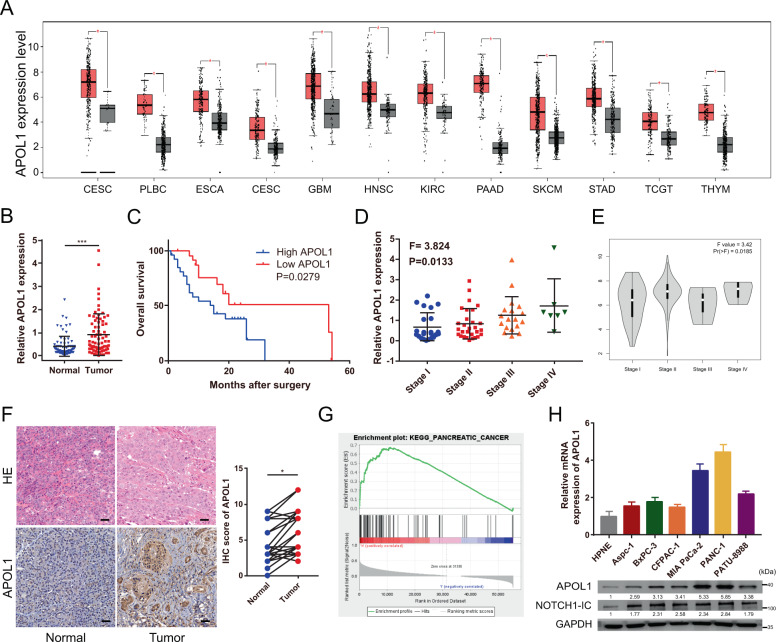
APOL1 is significantly upregulated in PC and is related to later pathological stage and poor prognosis. **A** APOL1 mRNA expression in different cancers. **B** APOL1 expression in 77 pairs of tumor tissues and adjacent normal tissues. **C** Prognostic analysis of APOL1 using survival data of 51 patients from our center. **D** APOL1 expression in patients divided by cancer stages using expression data from our center (*n* = 77). **E** APOL1 expression in patients divided by cancer stages using expression data from TCGA (*n* = 179). **F** IHC staining scores of APOL1 expression in 25 paired PC samples. Representative images of different APOL1 expression levels are shown in the left panel. Magnification: ×200. **G** GSEA results were plotted to visualize the correlation between the expression of APOL1 and dysregulated genes in PC. **H** APOL1 and NOTCH1-IC expression in PC cell lines (Aspc-1, Bxpc-3, CFPAC-1, MIA PaCa-2, PANC-1, and PATU-8988) compared with that in the normal pancreatic ductal epithelial cell line HPNE detected by qRT-PCR and western blotting. *P < 0.05; ***P < 0.001.

GEPIA revealed that APOL1 was upregulated in multiple cancers (Fig. 2A). Furthermore, using 77 pairs of postoperative specimens from our center, we validated that APOL1 expression was upregulated in PC specimens. Moreover, Kaplan–Meier survival analysis was conducted for 51 patients from our center. The results revealed that APOL1 expression was significantly associated with OS (*P* = 0.0279, Fig. 2C). Interestingly, we found that APOL1 was associated with advanced pathological stage at our center and in the TCGA database (Fig. 2D, E). Next, we analyzed the correlation between APOL1 and clinical data. APOL1 expression was significantly associated with pathological stage (*P* = 0.031), T stage (*P* = 0.041), lymph node metastasis (*P* = 0.019), and distant metastasis (*P* = 0.024) (Table 1). However, no correlation was observed between APOL1 and age or gender. Univariate analysis revealed that APOL1 expression, pathological stage, T stage, lymph node metastasis, and distant metastasis were also related to OS. Multivariate analysis further demonstrated that lymph node metastasis (hazard ratio (HR) = 2.614, 95% confidence interval (95% CI) 1.035–6.603, *P* = 0.042) and pathological stage (HR = 4.413, 95% CI 1.234–15.789, *P* = 0.022) were independent prognostic indicators for PC patients. Moreover, IHC staining was conducted to determine APOL1 protein expression in 25 PC tissues and paired non-cancerous tissues (Table 2). APOL1 protein expression levels were higher in PC compared with those in paired non-tumor tissues (Fig. 2F). GSEA also showed vital relationships between dysregulated gene expression in PC and APOL1 (Fig. 2G). APOL1 expression was upregulated in all six PC cell lines (Aspc-1, Bxpc-3, CFPAC-1, MIA PaCa-2, PANC-1, and PATU-8988) compared with that in the human pancreatic epithelial cell line HPNE. In addition, a western blotting against NOTCH1-IC in the cell lines was performed (Fig. 2H). The results demonstrated that NOTCH1-IC was significantly overexpressed at the protein levels of PC cell lines compared with HPNE, which may support the NOTCH1 signaling pathway activation in PC cells. These results suggested that APOL1 was significantly highly expressed in PC and is associated with poor prognosis.

**Table 1:** Correlations between APOL1 expression and clinical characteristics in PC patients.

Clinicopathologic parameters	Case (*n* = 77)	APOL1 expression		*P*-value
		Low	High	
Total	77	47	30	
Gender				0.267
Male	42	28	14	
Female	35	19	16	
Age				0.927
≥60	39	24	15	
<60	38	23	15	
Pathological stage				0.031
I	23	16	7	
II	29	21	8	
III–IV	25	10	15	
T stage				0.041
T1–2	35	17	18	
T3–4	42	30	12	
Lymph node metastasis				0.019
N0	31	22	9	
N1	34	22	12	
N2	12	3	9	
Distant metastasis				0.024
M0	70	46	24	
M1	7	1	6

**Table 2:** Univariate and multivariate analysis of clinic pathological factors for overall survival in PC patients.

Variables	Univariate analysis		Multivariate analysis	
	HR (95% CI)	*P*	HR (95% CI)	*P*
APOL1 (low vs. high)	2.458 (1.064–5.670)	0.035	1.915 (0.778–4.715)	0.158
Age (≥60 vs. <60)	0.918 (0.428–1.970)	0.826		
Gender (male vs. female)	0.941 (0.438–2.020)	0.876		
Pathological stage (I–II vs. III–IV)	2.397 (1.071–5.363)	0.033	1.348 (0.501–3.628)	0.555
T stage (T1–2 vs. T3–4)	1.220 (0.560–2.659)	0.617		
Lymph node metastasis (N0 vs. N1–2)	2.484 (1.000–6.172)	0.049	2.614 (1.035–6.603)	0.042
Distant metastasis (M0 vs. M1)	7.137 (2.617–19.467)	<0.001	4.413 (1.234–15.789)	0.022

### APOL1 promotes proliferation and inhibits apoptosis of PC in vitro

**Fig. 3:**
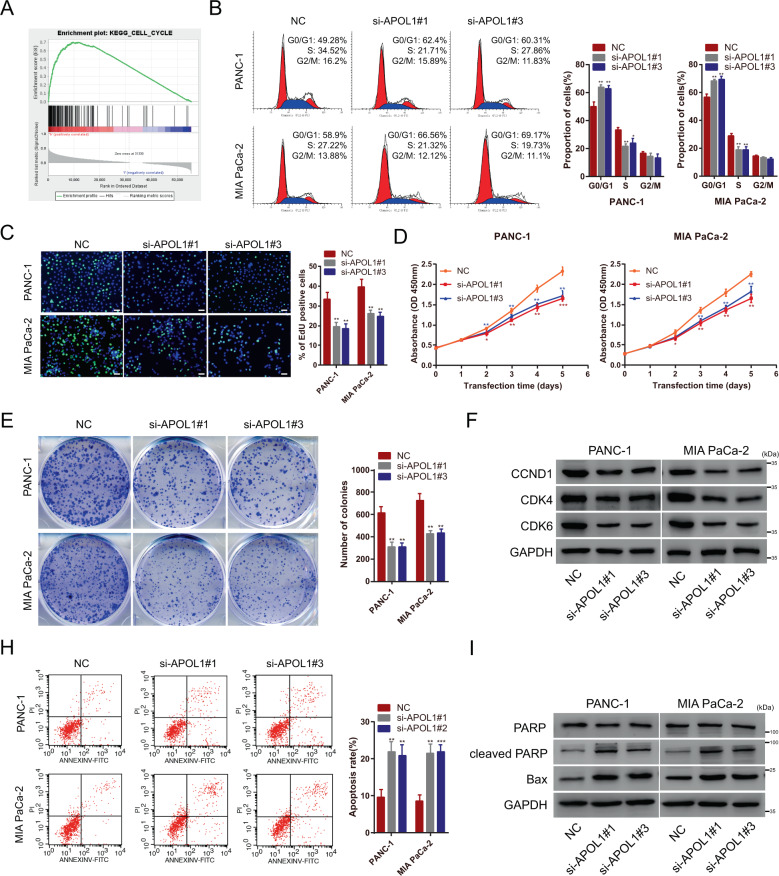
APOL1 promotes proliferation and inhibits apoptosis of pancreatic cancer cells in vitro. **A** GSEA results were plotted to visualize the correlation between the expression of APOL1 and cell cycle-related genes. **B** Flow cytometric cell cycle arrest assay was conducted to detect the proportion of PC cells after transfection of APOL1 siRNAs. **C** EdU assays were performed to detect the cell proliferative potential of APOL1 siRNA-transfected cells. **D** PANC-1 and MIA PaCa-2 were subjected to CCK-8 assays after transfection with APOL1 siRNAs. **E** PANC-1 and MIA PaCa-2 cells were placed in six-well plates after transfection with APOL1 siRNAs. The colony number was counted after 2 weeks. **F** Expression of cell cycle-related proteins was evaluated by western blotting after APOL1 silencing. **G** Flow cytometric apoptosis assay was used to detect the apoptosis rate of transfected PC cells. **H** Expression of apoptosis-related proteins PARP, cleaved PARP, and Bax was assessed by western blotting with the indicated treatment. Scale bar = 50 μm. *P < 0.05; **P < 0.01; ***P < 0.001.

Both APOL1 mRNA and protein levels were significantly elevated in pancreatic cells, especially in PANC-1 and MIA PaCa-2 cells, compared to those in HPNE cells. Therefore, to elucidate the function of APOL1 in cell behavior, we designed three independent siRNAs to knock down APOL1 expression in PANC-1 and MIA PaCa-2. APOL1 silencing was verified by quantitative reverse-transcriptase PCR and western blotting (Supplementary Fig. S1B). Using GSEA, significant relationships between cell cycle-related genes and APOL1 in PC were found (Fig. 3A). Based on GSEA results, we speculated that APOL1 may be involved in regulating PC cell proliferation. Therefore, flow cytometry, EdU, CCK-8, and colony formation assays were used to measure the effect of APOL1 on PC cell proliferation and growth. As speculated, APOL1 knockdown increased the proportion of cells in G0/G1 phase and decreased that in S phase (Fig. 3B), and significantly inhibited PC proliferation (Fig. 3C–E). Western blotting indicated that the cell cycle-related proteins CCND1, CDK4, and CDK6 were inhibited in si-APOL1-transfected PC cells (Fig. 3F). Next, we hypothesized that APOL1 may be involved in regulating apoptosis in PC. Thus, the effect of APOL1 on PC cell apoptosis was measured by flow cytometry apoptosis assay, which demonstrated that APOL1 knockdown significantly increased the apoptosis rate (Fig. 3H). Similarly, APOL1 knockdown upregulated the expression of the apoptosis-associated protein-cleaved PARP and Bax (Fig. 3I).

### APOL1 knockdown inhibited xenograft tumor growth

**Fig. 4:**
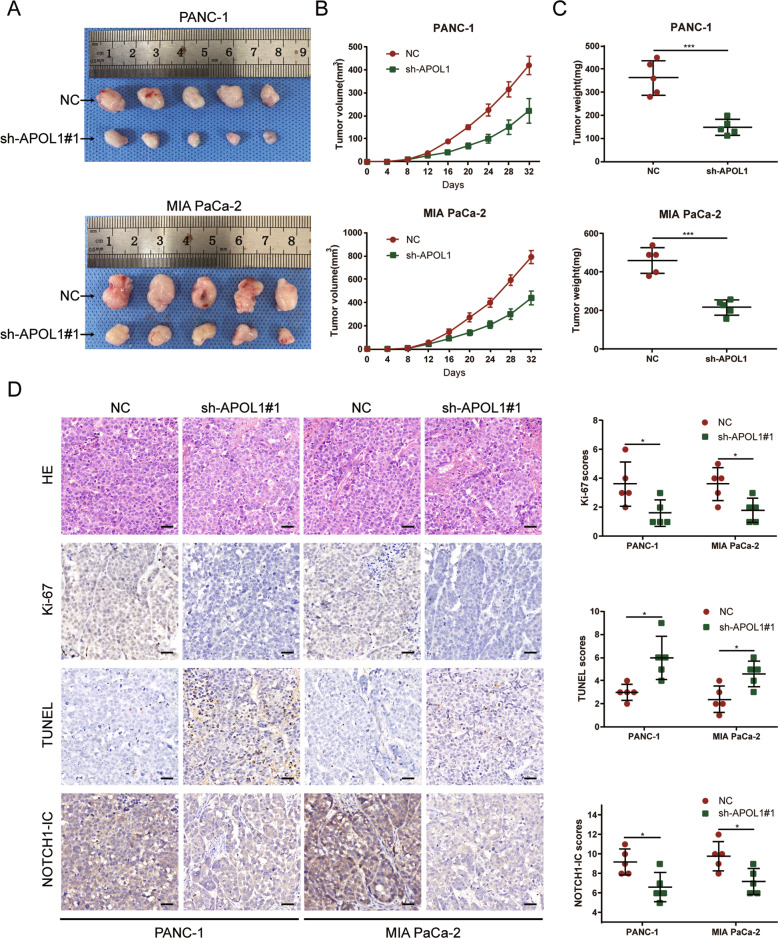
APOL1 knockdown inhibited xenograft tumor growth. **A** Photos of subcutaneous tumors. **B** The tumor volume was computed every 4 days after injection. **C** The tumor weights of subcutaneous xenografts. **D** Representative photographs of IHC staining of HE, ki-67, TUNEL, and NOTCH1-IC in xenograft tumors. Scale bar = 50 μm. *P < 0.05; ***P < 0.001.

To examine the effects of APOL1 on PC cell proliferation in vivo, PANC-1 and MIA PaCa-2 cells were transfected with sh-APOL1#1 or NC precursor sequence and subcutaneously injected into nude mice (*n* = 5 per group). After 32 days, mice were killed and whole tumors were excised. Tumor volume was measured every 4 days after injection. Tumors in the sh-APOL1#1 group were significantly smaller and weighed less than those in the NC group (Fig. 4A–C). To validate the ability of APOL1 to mediate tumor growth and apoptosis, xenograft tissues were stained with HE, ki-67, and TUNEL antibody for IHC analysis. The expression of ki-67, a proliferation marker, was decreased in sh-APOL1 PC tumors, whereas the apoptosis marker TUNEL was significantly increased. Likewise, these in vivo experiment results were consistent with in vitro findings that APOL1 knockdown inhibited tumor growth and induced apoptosis of PC cells (Fig. 4D).

### APOL1 exerts its function by activating NOTCH1 signaling pathway

**Fig. 5:**
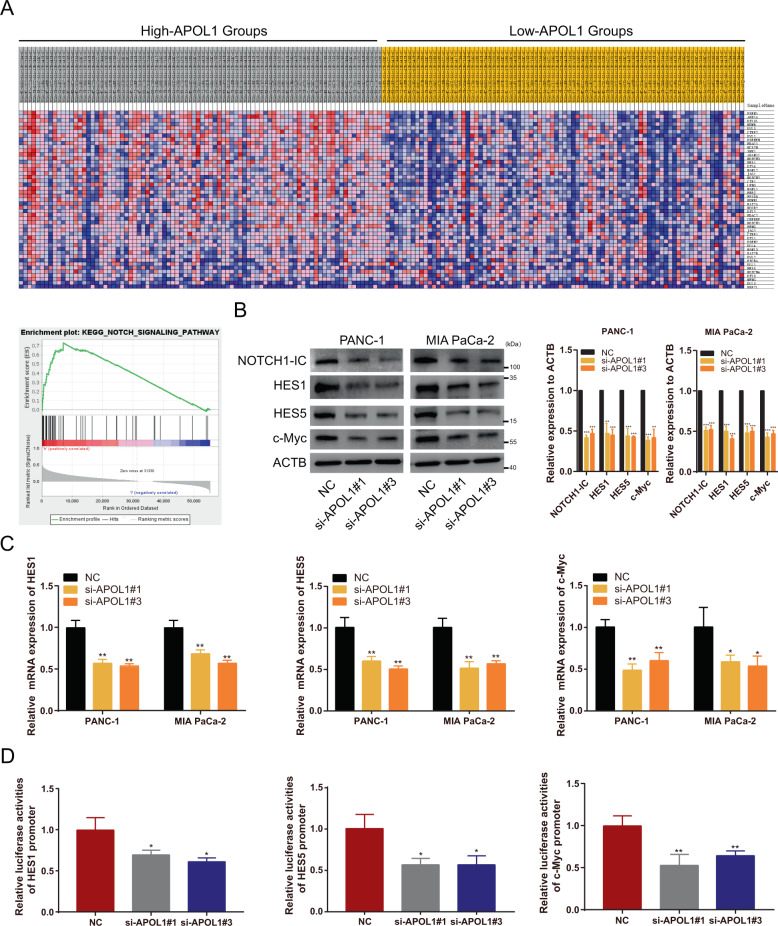
APOL1 exerts its function by activating NOTCH1 signaling pathway. **A** GSEA results were plotted to visualize the correlation between the expression of APOL1 and genes associated with the NOTCH signaling pathway. **B** The protein levels of NOTCH1-IC as well as its target genes, including *HES1*, *HES5*, and *c-Myc* were detected by western blotting after APOL1 silencing. **C** mRNA expression levels of HES1, HES5, and c-Myc were detected by qRT-PCR after APOL1 knockdown. **D** Luciferase activities were detected after co-transfected with si-APOL1 and indicated luciferase reporter plasmids in 293-T cells. *P < 0.05; **P < 0.01; ***P < 0.001.

To explore the underlying molecular mechanism of APOL1 function, we identified gene expression profiles from TCGA data (*n* = 179), grouping them as high- and low-APOL1 groups. The GSEA results demonstrated that APOL1 significantly enriched cancer-associated hallmark traits (Supplementary Tables S4 and S5), such as the NOTCH signaling pathway (Fig. 5A, B), P53 signaling pathway, ubiquitin-mediated proteolysis, and Toll-like receptor signaling pathway (Supplementary Fig. S2A–C). We hypothesized that APOL1 activates the NOTCH signaling pathway, because the latter had the highest enrichment score. To test this hypothesis, we performed western blotting, quantitative PCR, and dual-luciferase assays. We found that APOL1 silencing suppressed protein levels of NOTCH1-IC and its target genes (*HES1*, *HES5*, and *c-Myc*) (Fig. 5B). In addition, APOL1 depletion reduced mRNA levels of NOTCH1 targets (Fig. 5C). Further confirming the relationship between APOL1 and NOTCH1 signaling, dual-luciferase assays in 293-T cells verified the suppression of NOTCH1-IC transcriptional activities after APOL1 silencing (Fig. 5D). We also detected NOTCH1-IC expression in xenograft tumor tissues by IHC, which revealed downregulated NOTCH1-IC expression in the sh-APOL1#1 group (Fig. 4D). Collectively, these data indicate that APOL1 may exert its functions by enhancing NOTCH1 signaling pathways.

### APOL1 overexpression induced tumor-promoting effects in PC cells, which could be rescued by NOTCH1 knockdown

**Fig. 6:**
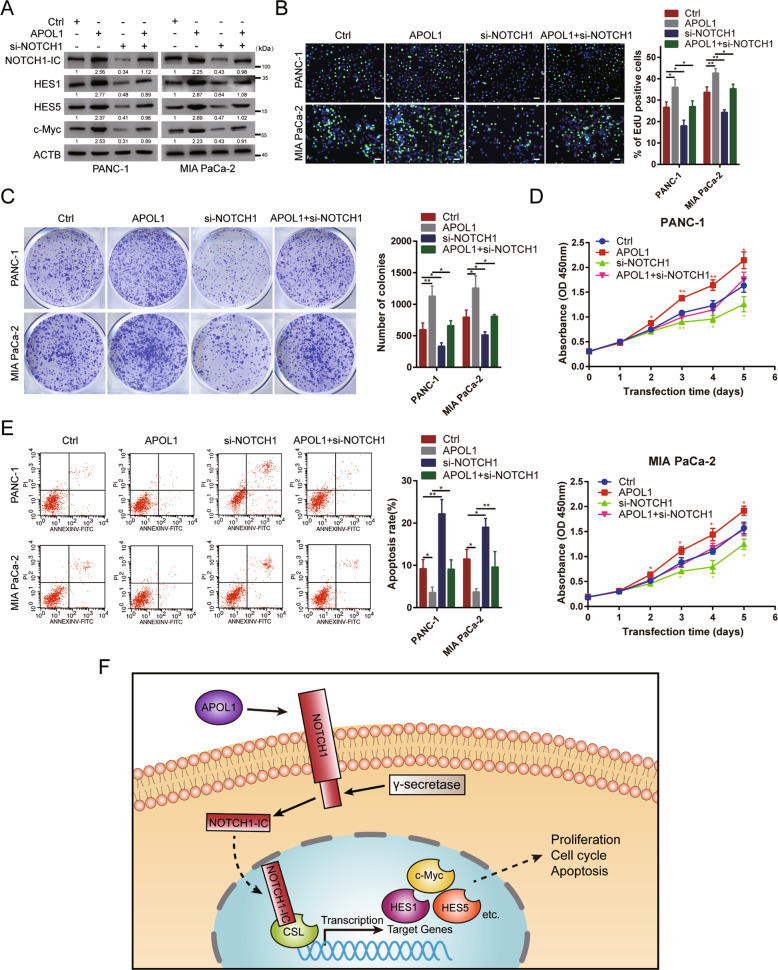
APOL1 overexpression induced tumor promotion in PC cells, which could be rescued by NOTCH1 silencing. **A** The expression of NOTCH1-IC, HES1, HES5, and c-Myc in PC cells co-transfected with pcDNA-APOL1 and si-NOTCH1. **B** EdU assay showed that NOTCH1 silencing abolished the increased EdU-positive rates of PC cells caused by APOL1 overexpression. **C** Colony formation assay and **D** CCK-8 assay demonstrated that NOTCH1 knockdown abolished the increased growth abilities of PC cells caused by APOL1 overexpression. **E** Flow cytometric apoptosis assay showed that NOTCH1 silencing abolished the decreased apoptosis rate of PC cells caused by APOL1 overexpression. **F** Proposed model demonstrating APOL1 could modulate proliferation, cell cycle, and apoptosis by activating NOTCH1 signaling pathway in PC. Scale bar = 50 μm. **P < 0.01; **P < 0.001.

To reveal the molecular mechanism underlying NOTCH1 modulation of APOL1 functions in PC cells, we co-transfected pcDNA-APOL1 and si-NOTCH1 into PANC-1 and MIA PaCa-2 cells. First, APOL1 overexpression upregulated NOTCH1-mediated proteins NOTCH1-IC, HES1, HES5, and c-Myc. When NOTCH1 was silenced, this upregulation was impaired (Fig. 6A). Next, we detected the effect of NOTCH1 knockdown on APOL1-induced promotion of proliferation in vitro. The EdU assay showed that NOTCH1 silencing could rescue the increase in EdU-positive cells induced by APOL1 overexpression (Fig. 6B). The colony formation assay indicated that NOTCH1 silencing could reverse the APOL1 overexpression-induced inhibition of proliferation (Fig. 6C). Moreover, the CCK-8 assay revealed that NOTCH1 silencing abolished the APOL1 overexpression-induced increase in PC cell proliferation (Fig. 6D). Furthermore, we examined the influence of NOTCH1 knockdown on APOL1-induced apoptosis inhibition. Silencing NOTCH1 impaired the APOL1 overexpression-mediated decrease in PC cell apoptosis (Fig. 6E). Collectively, these findings indicate that APOL1 serves as an oncogene by activating NOTCH1 signaling pathway in PC.

## Discussion

By analyzing the GSE41368, GSE28735, and GSE62452 datasets from the GEO database, we identified 146 common DEGs, including 106 upregulated and 40 downregulated DEGs. Futhermore, the PPI network and MCODE analysis showed that APOL1 is well situated as a network center and is linked with numerous other DEGs. Other hub genes, such as *CP*, *IGFBP5*, *LTBP1*, and *ALB*, were also linked with numerous genes, as reported previously [17, 29]. GO and KEGG analyses demonstrated that these DEGs are enriched in PC development-related pathways, such as the PI3K-AKT signaling pathway and ECM–receptor interaction. Moreover, the expression levels of the five hub DEGs were verified with GEPIA and the log-rank test for OS and DFS was conducted with Kaplan–Meier plotter. Interestingly, only APOL1 was associated with OS and DFS in PC patients. Therefore, we speculated that APOL1 may play a vital role in PC and is of great importance.

APOL1 belongs to the APOL gene family and acts as a minor component of HDL, and this gene family is associated with various cancers. In previous reports, APOL1 was considered to be highly elevated in various cancers, including PC [14–17]. However, APOL1 expression in PC is downregulated and can be used as a prognostic biomarker for patients with PC [30]. The expression of APOL1 in PC and its use as a prognostic biomarker are controversial. Thus, we confirmed that APOL1 was significantly upregulated in PC tissues obtained from our center, which was associated with later pathological stage, lymph node metastasis, and distant metastasis. Upregulated APOL1 was closely related to a short OS in PC patients, suggesting that APOL1 mRNA may be a novel diagnostic target. The effect of APOL1 on promoting PC was also verified in vivo and in vitro.

Proliferation and apoptosis are the two main characteristics of malignant tumor cells. Functionally, the potential role of APOL1 in PC cells was corroborated by flow cytometry of the cell cycle and apoptosis, EdU, CCK-8, and colony formation assays. Further investigation verified that knockdown of APOL1 observably inhibited cell growth and promoted cell cycle arrest and apoptosis in vitro, and inhibited cell proliferation in vivo, as evidenced by decreased tumor size in the PC mouse model. Western blotting further confirmed these results at the protein level. Based on these findings, we suggested that APOL1 is involved in PC tumorigenesis and development, and might act as a potential biomarker in PC.

For the mechanistic study, GSEA results were enriched in the NOTCH signaling pathway, which is a highly conserved cellular signal transduction system found in most multicellular organisms [31]. Four NOTCH receptors, NOTCH1–4, are found in mammal species. NOTCH receptors are single-transmembrane receptor proteins. They are heterooligomers comprising large extracellular parts, which interact with notch proteins containing a short extracellular domain, transmembrane domain, and small intracellular domain through calcium-dependent non-covalent interactions [32].

NOTCH1 is the most widely studied and characteristic member of the NOTCH receptor family due to its high mutation rate in human cancers [33]. Compared with non-tumor tissues, NOTCH1 was highly expressed in PC tissues and positively correlated with short-term survival, whereas knockdown of NOTCH1 inhibited cell proliferation and induced G1 cell cycle arrest, apoptosis, metastasis, and gemcitabine resistance in PC [34, 35].

Next, whether the oncogenic functions of APOL1 were dependent on regulating NOTCH1 signaling pathway was determined. We found that APOL1 was positively correlated with NOTCH1 signaling pathway and activation of NOTCH1 signaling pathway was inhibited by APOL1 knockdown (Fig. 6F). Finally, we showed that NOTCH1 knockdown could rescue the growth, cell cycle, and apoptosis of PC cells.

Our results strongly suggest the regulatory role of APOL1 and NOTCH1 signaling pathway in PC cell proliferation and apoptosis, which may provide a basis for future screening of PC biomarkers and selection of drug therapy targets. However, our experiment has some limitations; as in vivo and in vitro experiments were only conducted in the laboratory phase, additional data and further molecular mechanism research are needed before the results can be applied clinically in the future.

## Supplementary information

Supplementary figure legends

Table S1

Table S2

Table S3

Table S4

Table S5

Figure S1

Figure S2

## Data Availability

The datasets supporting the conclusions of this article are included within the article and its additional files.
