# Supplementary material for: Oncogene APOL1 promotes proliferation and inhibits apoptosis via activating NOTCH1 signaling pathway in pancreatic cancer
Source: Cell Death Dis. 2021 Aug 2;12(8):760. doi: 10.1038/s41419-021-03985-1 (PMC8329288; doi:10.1038/s41419-021-03985-1)
Supplement: Supplementary file 1 — Supplementary figure legends [file 41419_2021_3985_MOESM1_ESM.docx]

**Figure** **S1.** (A)The DEG PPI network was established via the STRING online database. (B) APOL1 expression was verified by qRT-PCR and western blotting after silencing of APOL1.

**Figure S2.** GSEA of APOL1. Hallmarks of GSEA enrichment results using APOL1 expression data from TCGA database.
