# Supplementary material for: Oncogene APOL1 promotes proliferation and inhibits apoptosis via activating NOTCH1 signaling pathway in pancreatic cancer
Source: Cell Death Dis. 2021 Aug 2;12(8):760. doi: 10.1038/s41419-021-03985-1 (PMC8329288; doi:10.1038/s41419-021-03985-1)
Supplement: Supplementary file 2 — Table S1 [file 41419_2021_3985_MOESM2_ESM.docx]

**Table S1. siRNA sequences used in this study**

| si-APOL1#1 sense | 5′-GGACAACCUUGCAAGACAATT-3′ |
| --- | --- |
| si-APOL1#1 anti-sense | 5′-UUGUCUUGCAAGGUUGUCCAG-3′ |
| si-APOL1#2 sense | 5′-CCAACUCACACGAGGCAUUTT-3′ |
| si-APOL1#2 anti-sense | 5′-AAUGCCUCGUGUGAGUUGGTA-3′ |
| si-APOL1#3 sense | 5′-GGAUUACCAGCAGUACCAUTT-3′ |
| si-APOL1#3 anti-sense | 5′-AUGGUACUGCUGGUAAUCCCG-3′ |
| si-NOTCH1 sense | 5′-GGGUGGUCAGGAAAAUCAUTT-3′ |
| si-NOTCH1 anti-sense | 5′-AUGAUUUUCCUGACCACCCTT-3′ |
| siRNA-NC sense | 5′-UUCUCCGAACGUGUCACGUTT-3′ |
| siRNA-NC anti-sense | 5′-ACGUGACACGUUCGGAGAATT-3′ |
