# Supplementary material for: Oncogene APOL1 promotes proliferation and inhibits apoptosis via activating NOTCH1 signaling pathway in pancreatic cancer
Source: Cell Death Dis. 2021 Aug 2;12(8):760. doi: 10.1038/s41419-021-03985-1 (PMC8329288; doi:10.1038/s41419-021-03985-1)
Supplement: Supplementary file 3 — Table S2 [file 41419_2021_3985_MOESM3_ESM.docx]

**Table S2** **Primer sequences used in this study**

| Primers used for qPCR: | |
| --- | --- |
| APOL1 forward | TGGACTACGGAAAGAAGTGGT |
| APOL1 reverse | CCTCCTTCAATTTGTCAAGGCTT |
| ACTB forward | CATGTACGTTGCTATCCAGGC |
| ACTB reverse | CTCCTTAATGTCACGCACGAT |
| HES1 forward | TCAACACGACACCGGATAAAC |
| HES1 reverse | GCCGCGAGCTATCTTTCTTCA |
| HES5 forward | CCGGTGGTGGAGAAGATG |
| HES5 reverse | GACAGCCATCTCCAGGATGT |
| c-Myc forward | CAAGAGGCGAACACACAACG |
| c-Myc reverse | GTCGTTTCCGCAACAAGTCC |
