# Supplementary material for: Oncogene APOL1 promotes proliferation and inhibits apoptosis via activating NOTCH1 signaling pathway in pancreatic cancer
Source: Cell Death Dis. 2021 Aug 2;12(8):760. doi: 10.1038/s41419-021-03985-1 (PMC8329288; doi:10.1038/s41419-021-03985-1)
Supplement: Supplementary file 4 — Table S3 [file 41419_2021_3985_MOESM4_ESM.docx]

| **Table S3. Primary and secondary antibodies.** | | | | |
| --- | --- | --- | --- | --- |
| **Antibody** | **Vendor** | **Cat. No.** | **Species** | **Dilution** |
| APOL1 | Proteintech | 11486-2-AP | Rabbit | 1:1000 |
| CCND1 | ABclonal | A0310 | Rabbit | 1:1000 |
| CDK4 | ABclonal | A11136 | Rabbit | 1:1000 |
| CDK6 | ABclonal | A0106 | Rabbit | 1:1000 |
| GAPDH | Proteintech | 60004-1-Ig | Mouse | 1:1000 |
| NOTCH1-IC | Cell signaling | Val1744 | Rabbit | 1:1000 |
| HES1 | Cell signaling | 11988 | Rabbit | 1:1000 |
| HES5 | ABclonal | A9768 | Rabbit | 1:1000 |
| c-Myc | Cell signaling | 18583 | Rabbit | 1:1000 |
| PARP | ABclonal | A19596 | Rabbit | 1:1000 |
| Bax | ABclonal | A19684 | Rabbit | 1:1000 |
| anti-mouse IgG (H+L), HRP conjugate | Proteintech | SA00001-1 | Goat | 1:5000 |
| Anti-Rabbit IgG(H+L), HRP conjugate | Proteintech | SA00001-2 | Goat | 1:5000 |
