# Supplementary figures and images for: Oncogene APOL1 promotes proliferation and inhibits apoptosis via activating NOTCH1 signaling pathway in pancreatic cancer
Source: Cell Death Dis. 2021 Aug 2;12(8):760. doi: 10.1038/s41419-021-03985-1 (PMC8329288; doi:10.1038/s41419-021-03985-1)

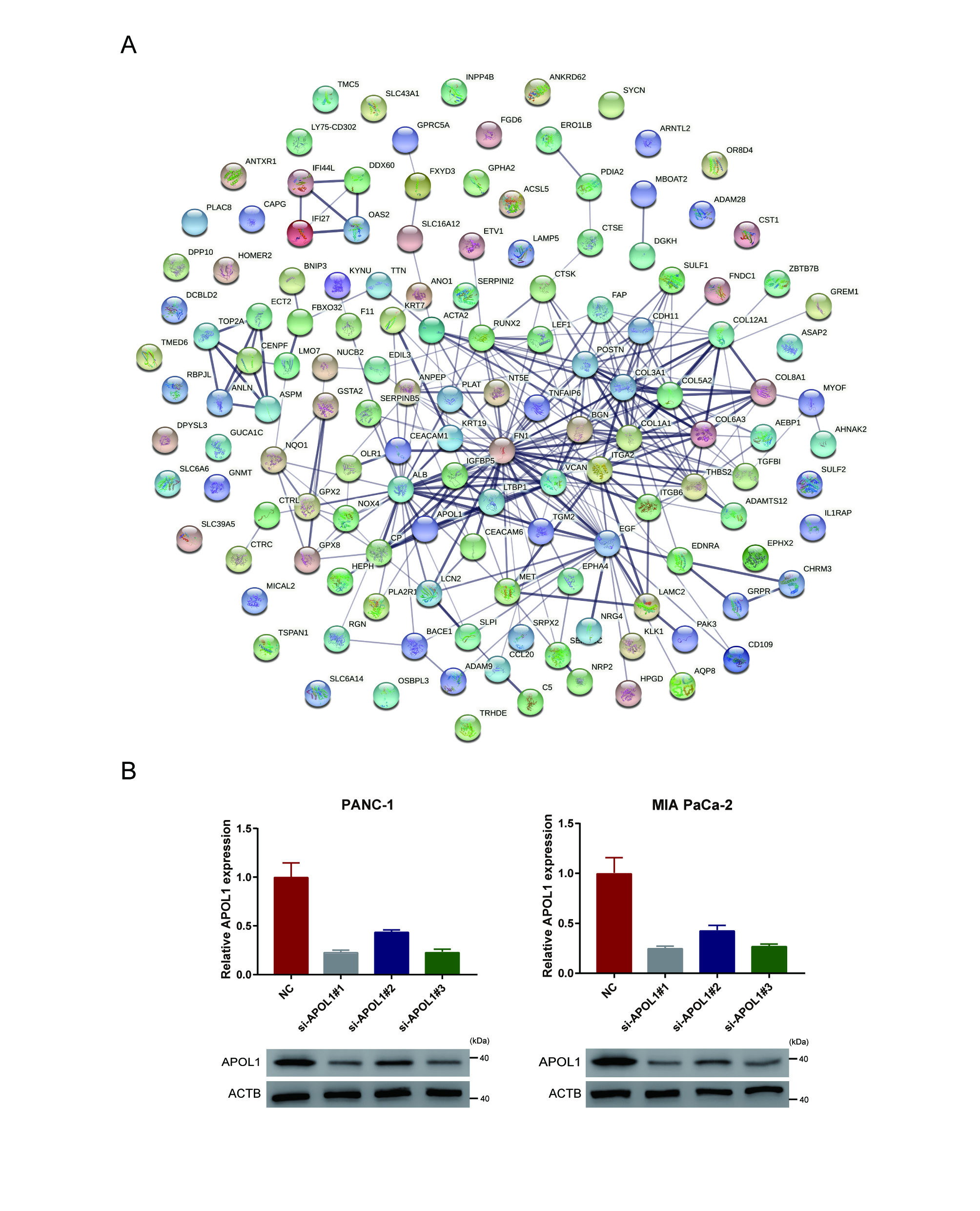

Supplement: Supplementary file 7 — Figure S1 [file 41419_2021_3985_MOESM7_ESM.tif]

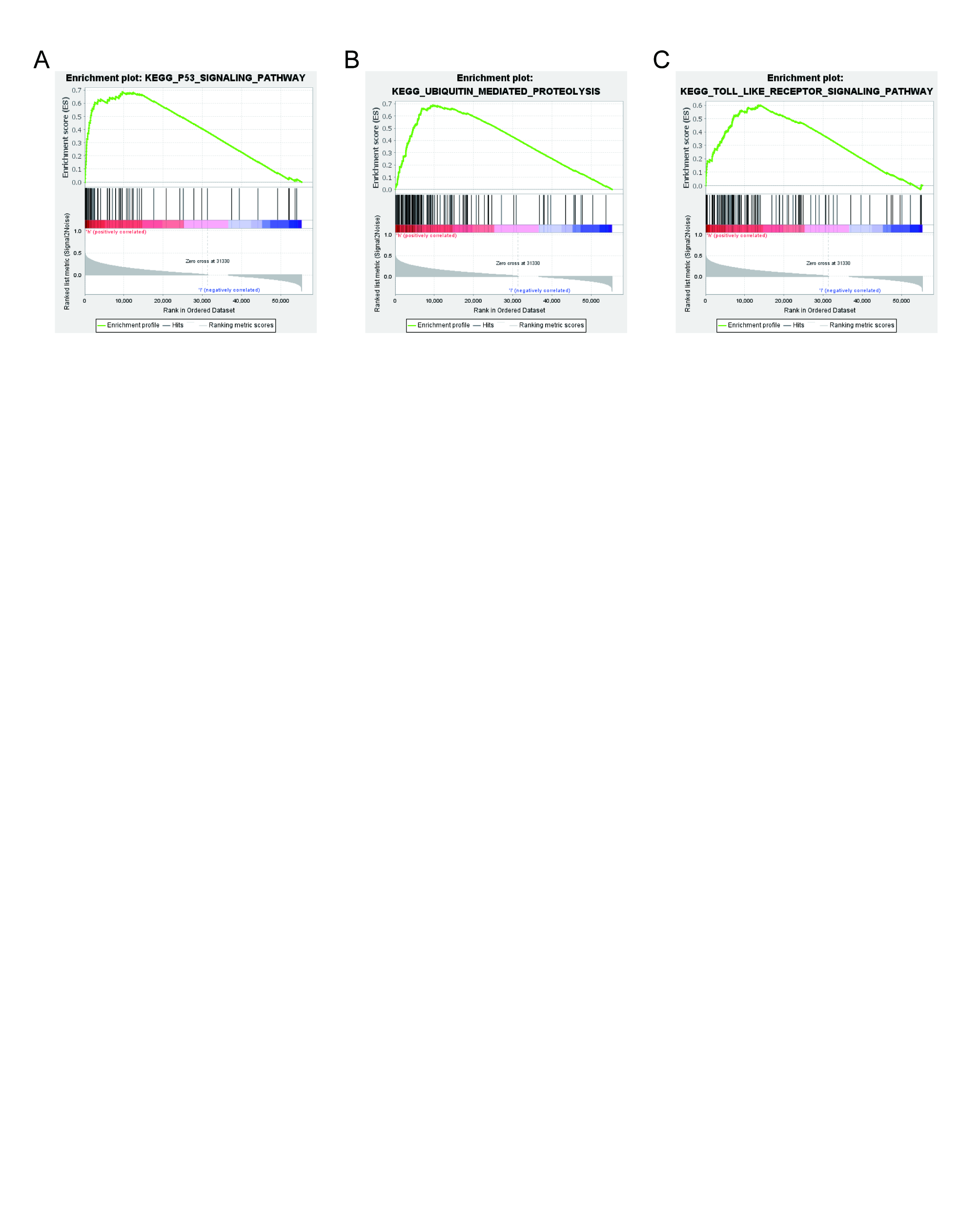

Supplement: Supplementary file 8 — Figure S2 [file 41419_2021_3985_MOESM8_ESM.tif]
